# Graphene-Modulated Removal Performance of Nitrogen and Phosphorus Pollutants in a Sequencing Batch *Chlorella* Reactor

**DOI:** 10.3390/ma11112181

**Published:** 2018-11-04

**Authors:** Gonghan Xia, Wenlai Xu, Qinglin Fang, Zishen Mou, Zhicheng Pan

**Affiliations:** 1 State Key Laboratory of Geohazard Prevention and Geoenvironment Protection, Chengdu University of Technology, Chengdu 610059, China; txgsfy@163.com (G.X.); nstxdy@163.com (Q.F.); 2 Haitian Water Grp. Co. Ltd., Chengdu 610059, China; pan12487616@126.com; 3 Department of Chemical Engineering, Tokyo University of Agriculture & Technology, Tokyo 1848588, Japan

**Keywords:** graphene, nitrogen and phosphorus removal, MDA, SOD, sequencing batch *Chlorella* reactor, SEM

## Abstract

In this work, the influence of graphene on nitrogen and phosphorus in a batch *Chlorella* reactor was studied. The impact of graphene on the removal performance of *Chlorella* was investigated in a home-built sewage treatment system with seven identical sequencing batch *Chlorella* reactors with graphene contents of 0 mg/L (T1), 0.05 mg/L (T2), 0.1 mg/L (T3), 0.2 mg/L (T4), 0.4 mg/L (T5), 0.8 mg/L (T6) and 10 mg/L (T7). The influence of graphene concentration and reaction time on the pollutant removal performance was studied. The malondialdehyde (MDA) and total superoxide dismutase (SOD) concentrations in each reactor were measured, and optical microscopy and scanning electron microscopy (SEM) characterizations were performed to determine the related mechanism. The results show that after 168 h, the total nitrogen (TN), ammonia nitrogen (AN) and total phosphorus (TP) removal rates of reactors T1–T7 become stable, and the TN, AN and TP removal rates were gradually reduced with increasing graphene concentration. At 96 h, the concentrations of both MDA and SOD in T1–T7 gradually increased as the graphene concentration increased. In optical microscopy and SEM measurements, it was found that graphene was adsorbed on the surface of *Chlorella*, and entered *Chlorella* cells, deforming and reducing *Chlorella*. Through the blood plate count method, we estimated an average *Chlorella* reduction of 16%. According to the water quality and microscopic experiments, it can be concluded that the addition of graphene causes oxidative damage to microalgae and destruction of the *Chlorella* cell wall and cell membrane, inhibiting the nitrogen and phosphorus removal in *Chlorella* reactors. This study provides theoretical and practical support for the safe use of graphene.

## 1. Introduction

Since Geim et al. used graphite to prepare graphene in 2004, scientists in various fields have extensively studied the physical, chemical, electrical, optical and mechanical properties of graphene [1]. Thanks to its special structure, high thermal conductivity, excellent electrical conductivity, high mechanical strength and unique optical properties, graphene can be widely used in the fields of composite materials, energy, catalysis, electronic devices, optical detection and environmental protection.

In the process of production, transportation, application, treatment and recovery, graphene will inevitably enter the environment. As a nanomaterial, microscale graphene can cause certain toxic effects on plants, animals and microorganisms [2]. If the functional microorganisms in the wastewater treatment process suffer from the toxic effect, the sewage treatment efficiency can be directly affected.

Using algae to treat wastewater is a hot topic in the environmental field. Chen Guang et al. used two cascade high-efficiency algal pond systems to treat rural sewage in Taihu, and showed that when the hydraulic retention time (HRT) was 8 days, the average removal rates of COD (chemical oxygen demand), TN and TP were 69.4%, 41.7% and 45.6%, respectively [3]. Huang et al. studied a high-efficiency algal pond system for treating rural sewage in Taihu. At HRT of 1.6 days, the effluent TN and TP can be kept at 5 mg/L and below 1 mg/L, respectively, which complies with the GB189182-2002 first level B emission standard of China [4]. Li et al. applied algal-immobilized methods to realize nitrogen and phosphorus removal in domestic sewage [5].

The influence of nanoparticles on algae has also been studied [6,7,8,9]. Li et al. studied the cell coercion of nano nickel oxide to *Chlorella vulgaris*, and found that the cell apoptosis phenomenon occurred when the cells were exposed to nano NiO [10]. Zhu et al. studied the toxic effects of fullerene (C_60_), single-walled carbon nanotubes (SWCNTs) and multiwalled carbon nanotubes on *Scenedesmus obliquus*. The results showed that the minimum inhibitory concentrations of the three materials for *Scenedesmus obliquus* were 5, 10 and 0.5 mg/L, respectively [11]. Xiao et al. carried out experiments on the effect of nano ZnO on phosphorus removal by *Chlorella*, and concluded that the addition of nano ZnO could inhibit the growth of *Chlorella*. The phosphorus removal efficiency of *Chlorella vulgaris* decreased from 76.2% to 27.4% in the first 7 h [12]. At present, the toxicity of nanoscale graphene to algae is mostly manifested in harmful effects on algae quantity, morphology and enzyme activity in vivo. The effect of graphene nanoparticles on nitrogen and phosphorus in activated sludge treatment systems has been widely studied. Nevertheless, there is almost no report on the effect on algae wastewater treatment systems. Therefore, the purpose of this paper is to reflect the effect of graphene nanoparticles on the removal of nitrogen and phosphorus from algae wastewater treatment systems, as well as to analyze the toxicity of graphene to algae from a novel point of view of wastewater treatment. Overall, in this work, the effect of graphene on a sequencing batch *Chlorella* reactor was studied.

*Chlorella pyrenoidosa* exhibits great efficiency in water treatment. In the experiments by Wang et al., the removal rates of nitrogen and COD in living sewage by *Chlorella* were 69% and 80.9%, respectively [13]. According to the study by Lu Furong and Huang Kui [14,15], the removal efficiency of nitrogen and phosphorus by *Chlorella* spp. could reach 70% and 60%, respectively, in the first 12 h under autotrophic conditions, and the organic compounds in a water body could be enriched and absorbed as assimilated carbon sources, nitrogen sources and sulfur sources during the growth and reproduction of *Chlorella* spp. Therefore, the timely addition of organic carbon will promote the absorption of nitrogen and phosphorus by *Chlorella*. By comparing the six species of algae, Cai et al. concluded that *Chlorella vulgaris* was suitable for nitrogen and phosphorus removal, and the removal rates of TP, TN and AN were 87.88%, 87.27% and 89.25%, respectively [16]. Through the test of five species of algae, Huang et al. concluded that *Chlorella* is the most suitable organism for sewage treatment. The removal rate of nitrogen and phosphorus in water can reach over 80% [17]. In this study, a home-built sequencing batch *Chlorella* reactor was employed, and *Chlorella vulgaris* was selected as the algae species. Graphene was added into the reactor. The effect of graphene concentration and reaction time on the wastewater treatment efficiency was studied, and the toxicity of graphene to the sequencing batch *Chlorella* reactor system was explored.

## 2. Materials and Methods

### 2.1. Experimental Materials

#### 2.1.1. Source of Graphene

Graphene was acquired from the xGnp Grade M graphene powder (American XGS Company, New York, NY, USA), which was stacked together by 6–10 layers of graphene sheets. The average thickness of the sheet is about 6–8 nm, and the specific surface area is 120–150 m^2^/g. The average diameter of the sheet is 5 µm.

#### 2.1.2. Source of *Chlorella*

The algae species were provided by the aquatic organisms of the Chinese Academy of Sciences (Beijing, China), which were cultured in a light incubator at 4000 lux, 23 °C, light/dark ratio of 12 h:12 h, and oscillating two times a day (8:00–9:00; 18:00–19:00). The BG (Blue-Green) 11 medium (Beijing Land Bridge Technology CO, LTD, Beijing, China) was used in the culture medium. The references of BG medium can be found in [18].

#### 2.1.3. Simulated Domestic Wastewater

In this experiment, artificial domestic sewage was prepared, and the water composition and its distribution were as the following (mg/L): C_6_H_12_O_6_ 150; peptone 150; CH_3_COONa 80; NH_4_Cl 80; KH_2_PO_4_ 26; MgSO_4_·7H_2_O 180; CaCl_2_ 10.6; NaHCO_3_ 80; EDTA 3; FeCl_3_·6H_2_O 0.45; MnCl_2_·6H_2_O 0.036; H_3_BO_3_ 0.045; ZnSO_4_·7H_2_O 0.036; CuSO_4_·5H_2_O 0.054; KI 0.054. The pH of the wastewater was adjusted to 8 [19].

### 2.2. Measurement Methods

According to the national standard method of China, TN was determined by alkaline potassium persulfate method, AN was determined by Nessler’s reagent photometry, and TP was obtained by ammonium molybdate spectrophotometric method. MDA and SOD indicators were purchased from Nanjing Jian Technology Co, Ltd (Nanjing, China).

### 2.3. Experimental Methods

**Figure 1:**
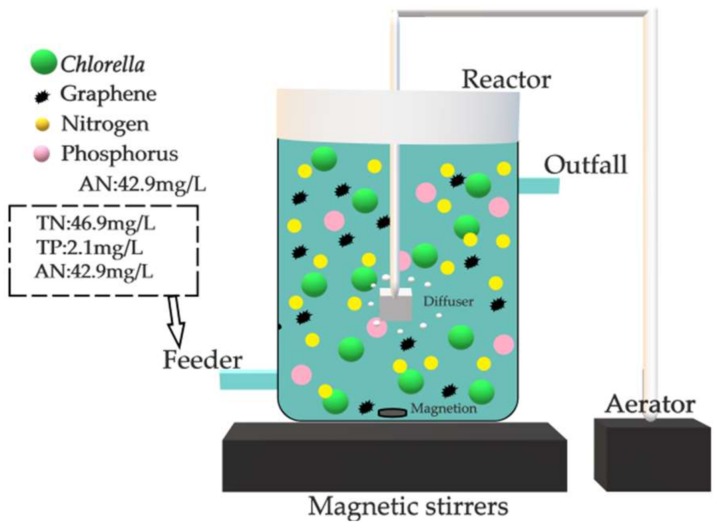
Schematic diagram of the reactor.

Seven identical sequencing batch *Chlorella* reactors T1, T2, T3, T4, T5, T6 and T7 were employed in the experiment. The volume of the reactors was 500 mL, as shown in Figure 1. Each reactor contained 250 mL artificial wastewater (high-temperature sterilization was used to avoid the influence of functional microorganisms in water). The number of initial *Chlorella* was 1.75 × 106. The TN content was 46.9 mg/L ± 0.1%, AN was 42.9 mg/L ± 0.2%, and TP was 2.1 mg/L ± 0.1%.

Graphene was added to each reactor, and the graphene concentration in each reactor is shown in Table 1.

According to the optimum growth conditions of *Chlorella vulgaris* [14], the reactor was placed in the light incubator at 4000 lux and 23 °C. 60 mL reaction liquid was taken every 12 h using a filter membrane suction method. Next, 60 mL artificial wastewater (high-temperature sterilization) with the same initial concentration was added to the reactor, and the TN, AN and TP were measured for each discharging water. The effect of graphene on removal efficiency of nitrogen and phosphorus pollutant in the sequencing batch *Chlorella* reactor was investigated.

### 2.4. Oxidative Stress Experiment

Based on the water quality experiment, the concentration of MDA and the activity of SOD in each reactor were determined. 

MDA is an important indicator of cell oxidative damage. It mainly reflects the concentration of oxygen free radicals produced by the interaction among phospholipids, enzymes and fatty acids in membrane receptors. Higher MDA concentration leads to greater lipid peroxidation in plant cells, which indirectly reflects the degree of damage to the organism. The vitality of SOD reflects the ability of the organism to scavenge oxygen free radicals, and increasing SOD activity indicates the increase of oxygen free radicals in plant cells. Therefore, SOD indicators often match the MDA index with the oxidative damage of reactive cells.

The MDA kit is developed by Nanjing Jian Cheng Technology Co, Ltd (Nanjing, China). The test method is as follows: 

The reagent and the sample were added to the test kit. The test tubes include blank tube (OD_0_), standard tube (OD_s_), measuring tube (OD_m_) and the care (OD_c_). The vortex mixer of each sample was mixed, and the tube mouth was tightened with fresh-keeping film. A small hole was opened with needle, and the water bath was set to 95 °C for 40 min. After being taken out of the water, the sample was cooled, followed by centrifugation for ten minutes at 3500–4000 turns/points. The absorbance of the supernatant for each tube was measured at 523 nm and 1 cm light path. Double water was used to calibrate zero point.

The formula for calculating MDA content in plant tissues is as below (1):

(1) $$\mathrm{MDA}=\;\frac{{\mathrm{OD}}_{m}-{\mathrm{OD}}_{c}}{{\mathrm{OD}}_{s}-{\mathrm{OD}}_{0}}\times 10\;\mathrm{nmol}/\mathrm{mL}\;÷C\left(\mathrm{protein}\right)$$

The SOD kit is developed by Nanjing Technology Co, Ltd (Nanjing, China). The test method is as follows: 

According to the reagent box operation and samples, the test tube (OD_m_) and the sample (OD_c_) were used. The vortex mixer of each sample was mixed and placed at 37 °C for 40 min in water bath with constant temperature. Next, the chromogenic agent was added to the mix, and placed at room temperature for ten minutes. The absorbance of the supernatant of each tube was measured at 550 nm and 1 cm light path with double water to adjust zero.

The formula for calculating the total SOD vitality is shown in Equation (2):

(2) $$\mathrm{SOD}=\frac{{\mathrm{OD}}_{c}-{\mathrm{OD}}_{m}}{{\mathrm{OD}}_{c}}÷50\%\times \frac{V_{\mathrm{total}}}{V_{\mathrm{sampling}}}÷C\left(\mathrm{protein}\right)$$

## 3. Results and Discussions

### 3.1. Graphene Characterization

**Figure 2:**
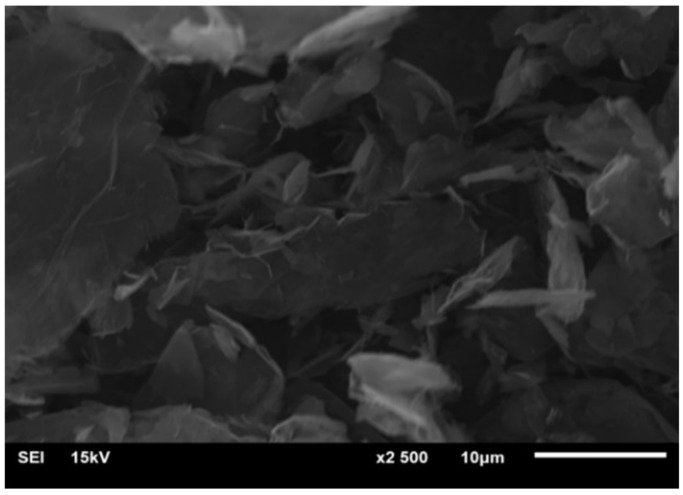
SEM characterization of graphene sheets.

Graphene characterization is shown in Figure 2.

### 3.2. Effect of Graphene Content and Reaction Time on Removal Efficiency of Pollutants in Reactor

#### 3.2.1. Treatment Effect of TN in Reactors T1–T7

**Figure 3:**
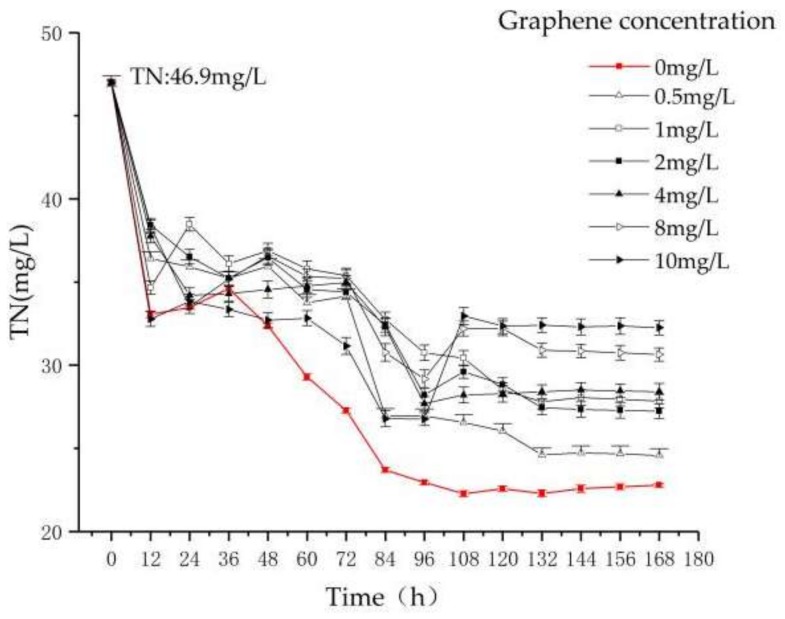
The variation of TN as a function of reaction time and graphene concentration.

From Figure 3, it can be seen that the removal efficiency of TN in T1 (the concentration of graphene is 0 mg/L) increases gradually at 0–96 h. After 96 h, the removal rate of TN tends to be stable at 51.7% ± 0.3%.

At 0–96 h, the removal rate of TN increases with different graphene concentrations in T2–T7. At 12 h, the removal efficiency of T7 (the concentration of graphene is 10 mg/L) is greater than T1, indicating that graphene has adsorption on TN. After 96 h, the TN concentrations of reactors T4, T5, T6 and T7 increase, and the TN concentrations in reactors T2 and T3 become stable, which indicates that the capacity of the reactor to remove TN decreases with increasing graphene concentration. Finally, the TN removal rates of T2–T7 are stable at 47.7% ± 0.1%, 42.9% ± 0.3%, 41.5% ± 0.4%, 39.6% ± 0.2%, 34.9% ± 0.1%, and 30.8% ± 0.3%, respectively. In this experiment, the removal efficiency of TN in the sequencing batch *Chlorella* reactor decreases as the concentration of graphene increases.

#### 3.2.2. Treatment Effect of AN in Reactors T1–T7

**Figure 4:**
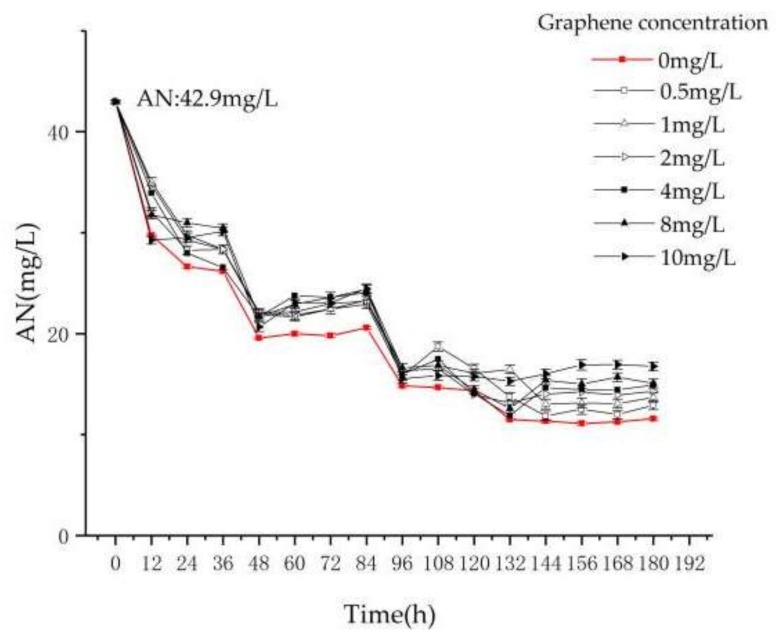
The variation of AN with reaction time and graphene concentration.

From Figure 4, the AN removal in each reactor is similar to TN, indicating that the removal of nitrogen in the sequencing batch *Chlorella* reactor is mainly due to the removal of AN. In T1, the removal rate of AN tends to be stable at 73.2% ± 0.4%.

In T2–T7, the removal rate of AN by T7 in the first 12 h is greater than T1, which proves that graphene has adsorption effect on AN in water. However, with increasing time, the adsorption of graphene in reactor T7 becomes gradually stable, and the removal of AN mainly depends on the assimilation of *Chlorella* to AN and the volatilization of AN itself [20,21,22]. At 0–121 h, the concentrations of AN in reactors T1–T7 are decreasing due to volatilization and *Chlorella* assimilation. After 121 h, the gradual increase of graphene concentration in T2–T7 increases the destruction of *Chlorella*. Finally, the removal efficiencies of AN in T2–T7 are stable at 70% ± 0.5%, 68.3% ± 0.2%, 66.9% ± 0.2%, 65.4% ± 0.1%, 65.1% ± 0.4%, and 61.2% ± 0.3%, respectively. In this experiment, the removal efficiency of AN in the sequencing batch *Chlorella* reactor decreases as the concentration of graphene increases.

#### 3.2.3. Treatment Effect of TP in Reactors T1–T7

**Figure 5:**
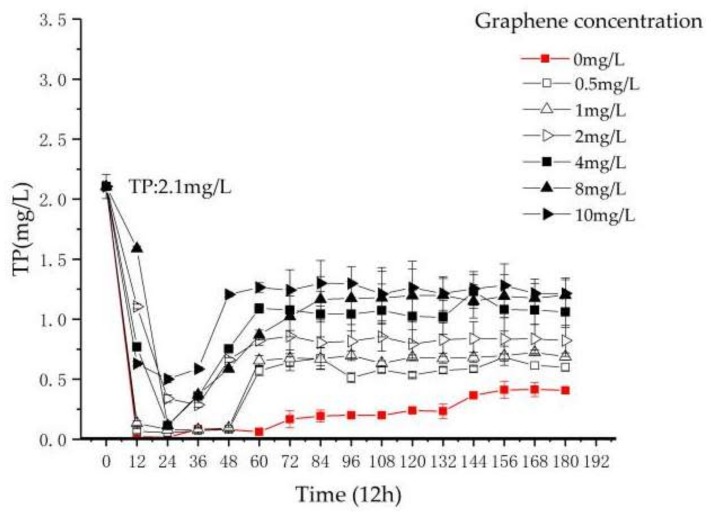
The variation of TP with reaction time and graphene concentration.

From Figure 5, graphene shows great influence on TP in the reactor. In T1, the removal efficiency of TP is stable at 80.6% ± 0.8%.

At 0–36 h, the TP concentration in T2–T7 decreases continuously. After 36 h, the TP concentration in T2–T7 increases continuously. The TP concentrations in T2–T7 after 168 h are rising gradually. The removal efficiencies of TP in T2–T7 are stable at 71.4% ± 0.3%, 67.3% ± 0.2%, 61% ± 0.1%, 49.6% ± 0.1%, 43% ± 0.3% and 42.3% ± 0.3%, respectively.

In previous studies, the destruction of the algal cell wall and cell membrane by nanoparticles was observed. The increase of TP in the reactor containing graphene is due to the damage by graphene to the cell wall and cell membrane of *Chlorella*. The reduction of *Chlorella* quantity reduced the phosphorus removal efficiency, and the decomposition of *Chlorella vulgaris* itself increases the phosphorus content in the process. This result is consistent with the research by Xiao et al. on phosphorus removal in a nano-ZnO-treated *Chlorella* treatment system [12].

To sum up, in the determination of TN, AN and TP in the reactor, the addition of graphene inhibited the removal of nitrogen and phosphorus pollutants in the reactor. On one hand, the photosynthesis of *Chlorella vulgaris* may be affected by the shielding effect of graphene. On the other hand, there may be a toxic effect by graphene as a nanoparticle on *Chlorella*, leading to *Chlorella* damage, decreasing its quantity and self-decomposition. Therefore, the nitrogen and phosphorus in water cannot be effectively removed.

### 3.3. Oxidative Stress Experiment

In the experiment of water quality test for T1–T7, we can conclude that the increase of graphene concentration decreases the efficiency of *Chlorella* in nitrogen and phosphorus removal. In order to further verify the effect of graphene on the damage and reduction of *Chlorella*, the MDA content and SOD activity in the reactor were measured, and the *Chlorella* was observed by high-magnification optical microscopy and SEM.

#### 3.3.1. Result of MDA Measurements

Cells can produce oxygen free radicals by enzyme systems and nonenzyme systems. Oxygen free radicals can cause cell damage by peroxidation of polyunsaturated fatty acids in biofilm, triggering lipid peroxidation and forming lipid peroxides. MDA is a product of lipid peroxidation. MDA can be determined by lipid peroxidation. It reflects the degree of lipid peroxidation and indirectly reflects the extent of cell damage.

Combined with the above pollutant removal experiments, the water samples from each reactor were used to determine the concentration of MDA at 96 h [14].

**Figure 6:**
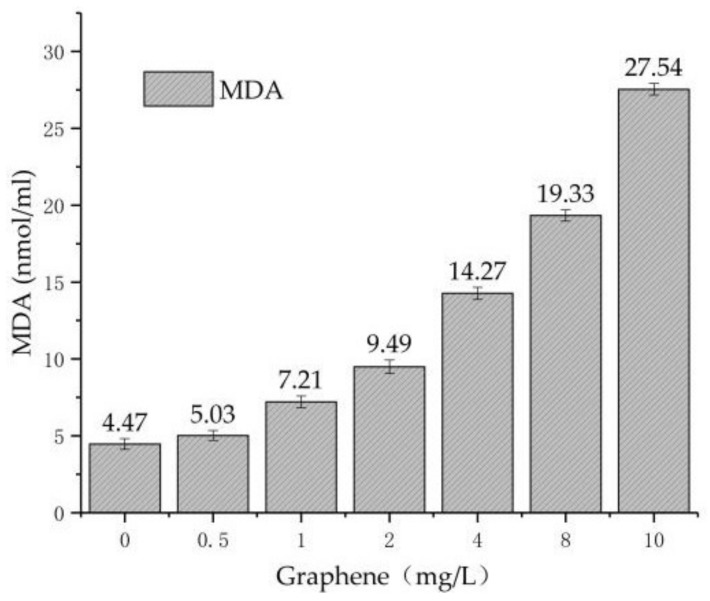
The variation of MDA with graphene concentration.

From Figure 6, at 0–96 h, with the increase of graphene concentration in reactors T1–T7, the concentrations of MDA increase. Compared to T1, the increase of MDA in T2 is not obvious. This indicates that the oxidation damage of *Chlorella* is not obvious at low graphene concentration. Nevertheless, after increasing the concentration of graphene, the increase of MDA becomes significant. In T7, the MDA concentration of 27.54 nmol/mL is 6.16-times that of T1, which proves that graphene can cause heavy damage to *Chlorella* cells.

#### 3.3.2. Results of SOD Determination

SOD plays an important role in the oxidation and antioxidant balance of the body. SOD can clear the superoxide anion radical, and protect the cell from damage. High SOD activity indicates high free-radical content and high degree of cell damage.

**Figure 7:**
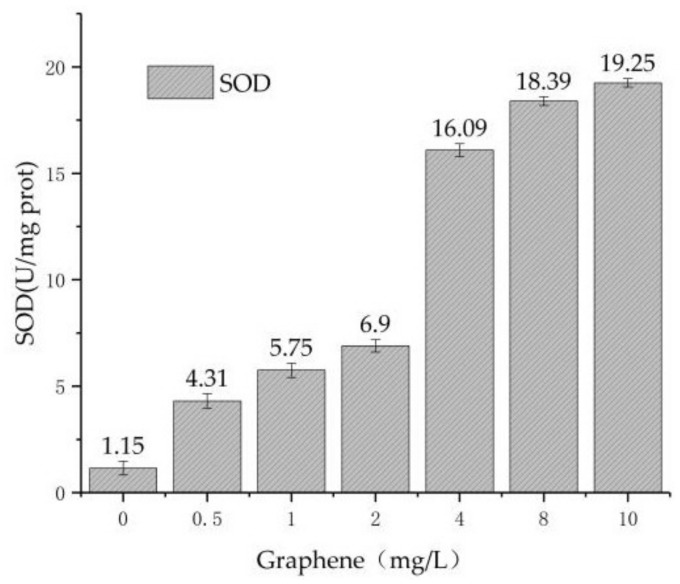
The variation of SOD with graphene concentration.

Combined with the above pollutant removal experiments, 96 h was used as the time to determine the SOD concentration [10]. From Figure 7, in T1–T7, the concentration of SOD increases with the increase of graphene concentration, from the initial 1.15 μ/mg prot to 19.25 μ/mg prot. The addition of graphene induces the increase of SOD activity in *Chlorella*, which is also related to the increase of MDA. The corresponding relationship between SOD and MDA proves that graphene produces oxidative stress on *Chlorella*. The research [22] by Zhao shows that the nanoparticles can combine with the algae chloroplast to cause lipid peroxidation of the algae cell membrane. The result is consistent with our study.

#### 3.3.3. Observation by Optical Microscope

**Figure 8:**
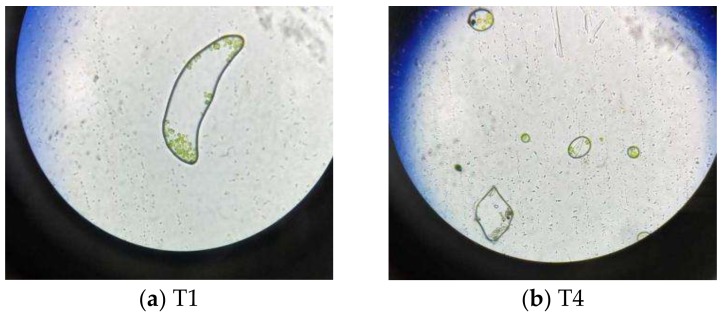
(**a**) *Chlorella* vulgaris under normal conditions; (**b**) *Chlorella* in reactor T4 with graphene addition.

Through the test of MDA concentration and SOD activity, the damage effect of graphene to the algal cells in reactor T4 is apparent. Therefore, the *Chlorella* from T1 and T4 were examined under a 40× optical microscope. It can be seen from Figure 8 that the morphology and internal structure of *Chlorella vulgaris* under normal conditions (T1) and with added graphene (T4) demonstrate significant change under microscope. Figure 8a shows that the *Chlorella* in T1 is striped or spherical with the basic structure of microorganisms inside the cell, such as chloroplasts. Figure 8b shows that there are some black materials in the microalgal algae. From the image, we can see that graphene enters the *Chlorella* cell, and affects the chloroplast inside the *Chlorella*. Combining with previous experiments on the coexistence of *Chlorella* and graphene, the experiments show that graphene exerts a harmful effect on the growth of *Chlorella*. The result [23] is consistent with our study.

#### 3.3.4. SEM Measurements

**Figure 9:**
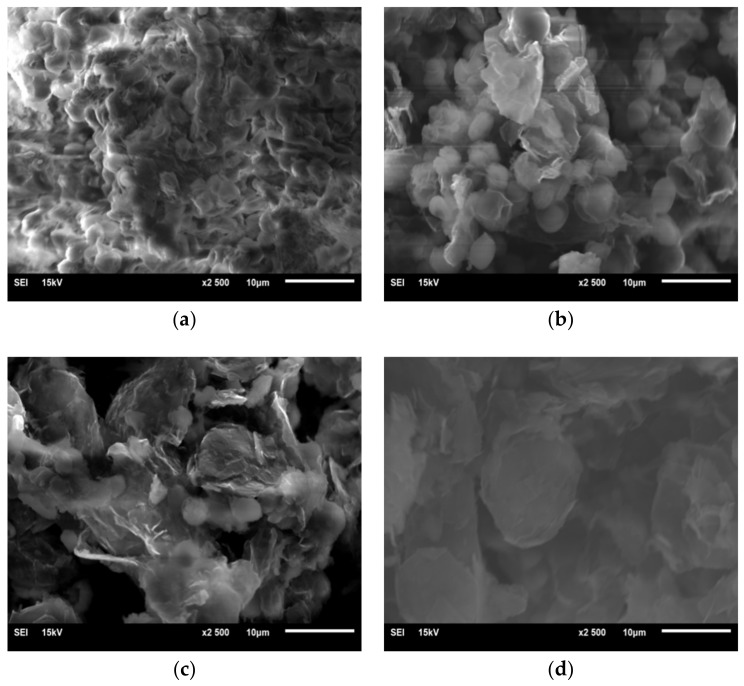
(**a**) SEM image of *Chlorella* in normal state; (**b**) SEM image of *Chlorella* in reactor T3 with graphene concentration of 1 mg/L; (**c**) SEM image of *Chlorella* in reactor T5 with graphene concentration of 4 mg/L; (**d**) SEM image of *Chlorella* in reactor T6 with graphene concentration 8 mg/L.

In order to further obtain morphological changes of *Chlorella* with increasing graphene concentration, *Chlorella vulgaris* in T1, T3, T5 and T6 reactors was observed under SEM. From Figure 9, *Chlorella* in T1 has a smooth surface with regular morphological algal bloom (Figure 9a). When graphene is introduce in T3, it forms flocs around the *Chlorella*. It is speculated that the addition of graphene causes self-protection of *Chlorella*, resulting in increased extracellular polymeric substances on the surface of *Chlorella* (Figure 9b). The increase of graphene and decrease in the number of *Chlorella* can be seen in T5 (Figure 9c). The morphology of *Chlorella* in reactor T5 is irregular. The white flocculus surface of *Chlorella* is surrounded by *Chlorella*, and *Chlorella* demonstrates an irregular smog-ball shape (Figure 9c). From the *Chlorella* in T6 (Figure 9d), complete *Chlorella* almost disappears, and the surface of *Chlorella* is covered by graphene. The full contact between graphene and *Chlorella* causes the morphology of *Chlorella* to become extremely irregular, and the white floc on the surface of *Chlorella* disappears. Instead of white floc, flaky graphene, attached to the surface of *Chlorella*, damages the *Chlorella*.

Using the blood plate count method, we obtained *Chlorella* with an average reduction of 16%.

## 4. Conclusions

(1) In the experiment of pollutant removal, the removal rates of TN and AN of reactors T1–T7 decrease with the increase of graphene concentration, and the removal efficiency of TN in reactor T2–T7 is lower than T1 by 4%, 8.8%, 10.2%, 12.1%, 16.8% and 20.9%, respectively. The removal rate of AN is lower by 10.2%, 16.5%, 20.2%, 21.2%, 24% and 28.4%, respectively. The removal rate of TP is lower by 9.2%, 13.3%, 19.6%, 31%, 37.6% and 38.3%, respectively.
(2) In order to verify the damage of *Chlorella* by graphene, the concentration of MDA and the activity of SOD in the algal cells were measured. The results show that in reactors T1–T7, the concentration of MDA increases from 4.47 nmol/mL to 27.54 nmol/mL, and the activity of SOD increases from 1.15 μ/mg prot to 19.25 μ/mg prot. As the concentration of graphene increases, MDA and SOD increase regularly during the same period. Thus, it is concluded that the addition of graphene can cause oxidative damage to *Chlorella vulgaris*.
(3) Using optical microscopy and SEM, it is found that graphene is adsorbed on the surface of *Chlorella*, and enters the interior of *Chlorella*. It is shown that graphene causes microscopic damage on *Chlorella*. Through the blood plate count method, we estimated an average *Chlorella* reduction of 16%. Nevertheless, it is necessary to further explore whether graphene destroys or modifies the internal structure and material of chloroplasts inside *Chlorella*.
(4) The damage of *Chlorella* by graphene can inhibit *Chlorella* from removing pollutants in sewage, and decrease the removal efficiency of nitrogen and phosphorus pollutants in the sequencing batch *Chlorella* reactor. This study provides theoretical and practical support for the safe use of graphene.

## Figures and Tables

**Table 1:** Graphene concentration in each reactor.

	T1	T2	T3	T4	T5	T6	T7
Graphene (mg/L)	0	0.5	1	2	4	8	10
